# Hochschulpolitischer Aktivismus in der Wissenschafts‑, Medizin- und Technikgeschichte: die AG-Mittelbau

**DOI:** 10.1007/s00048-024-00395-0

**Published:** 2024-07-12

**Authors:** Bettina Bock von Wülfingen, David Freis, Nadine Metzger, Christian Sammer, Alexander von Schwerin, Heiko Stoff, Florence Vienne, das AG-Mittelbau-Kollektiv

**Affiliations:** Institut für Pharmaziegeschichte, Technische Universität Braunschweig, Beethovenstr. 55, 38106 Braunschweig, Deutschland

In den 2010er Jahren entwickelte sich der Jour fixe von Bettina Wahrig an der TU Braunschweig zu einem wichtigen Treffpunkt einiger Promovierender und Postdocs aus dem Bereich der Wissenschafts- und Pharmaziegeschichte. Dabei ging es nicht nur um die Diskussion laufender Projekte, sondern auch um den Zustand und die Zukunft unseres Fachs. Bettina Wahrig hielt die Abteilung regelmäßig mit der Erstellung einer Übersicht über Institutionen, Personalsituation und Publikationen im Bereich der Wissenschaftsgeschichte für das Deutsche Nationalkomitee der International Union for the History and Philosophy of Science (IUHPS) in Atem. An einem grauen Herbsttag im Jahr 2011 forderte schließlich Florence Vienne anlässlich einer erhitzten Debatte über die prekäre Situation des Mittelbaus, dass wir Postdocs selbst die Initiative zur Verbesserung unserer Lage ergreifen müssten. Der Geist französischer Arbeitskampfkultur war ansteckend, und so begannen wir, eine programmatische Schrift zu formulieren, die 2014 mit dem Titel „Für die Überwindung des deutschen Sonderwegs der Nachwuchsuniversität“ als Positionspapier der „Arbeitsgruppe Mittelbau der Medizin‑, Technik- und Wissenschaftsgeschichte“ kursierte.

Eine Kernaussage darin lautete, dass es sich beim Mittelbau eben nicht um „Nachwuchs“, sondern um die „Basis“ an den Hochschulen handle. Wir kritisierten die im deutschen Hochschulsystem widersinnig festgeschriebenen Karrierewege, die einzig auf die Professur zulaufen, alle anderen Positionen größtenteils in der Befristung halten und die Inhaber:innen als „Nachwuchs“ abqualifizieren. Dem Mittelbau würden so zwar akademische Pflichten aufgebürdet, nicht aber eine akademische Zukunft zugebilligt. Die Situation in den uns betreffenden Fächern erwies sich als doppelt problematisch, da diese im Wissenschaftssystem insgesamt eine Abwertung erfuhren: Professuren wurden abgebaut, was sich systematisch negativ auf die Situation des Mittelbaus auswirkte. Ein Faktor war dabei die Einführung des Querschnittsbereichs „Geschichte, Theorie und Ethik der Medizin“ im Medizinstudium ab 2003, die dazu führte, dass in den folgenden Jahren viele Professuren für Medizingeschichte bei der Neubesetzung an Medizinethiker:innen vergeben wurden. Deshalb war und ist es auch ein Anliegen der Arbeitsgruppe, die gesellschaftliche Bedeutung dieser kleinen Fächer hervorzuheben.

Das Positionspapier formulierte fünf grundsätzliche Forderungen: 1. Angleichung der Ausgaben für Bildung und Forschung an die in den europäischen Nachbarländern üblichen anteiligen Ausgaben; 2. Gleichberechtigte Mitbestimmung (volle Mitgliedschaft von Promovierenden und Habilitierenden) in den Universitätsgremien; 3. Rückkehr zu Hochschulstrukturen, die primär auf dem Prinzip der Festanstellung statt auf mithilfe von Drittmitteln finanzierten befristeten Stellen basieren (Verbesserung der Grundausstattung); 4. Gleiche Chancen; 5. Familiengerechte Arbeitsbedingungen. Diese Kernforderungen wurden durch zwölf Punkte ergänzt, zu denen etwa die Schaffung zusätzlicher unbefristeter Mittelbaustellen neben der Professur, Mindeststandards für befristete Anstellungsverhältnisse und die automatische Entfristung ohne Wissenschaftszeitvertragsgesetz nach mehrjährigen befristeten Anstellungszeiten gehörten. Damit sollte Anschluss an Ideen zur Neugestaltung des Hochschulsystems insgesamt gefunden werden, wie sie fünfzig Jahre zuvor formuliert worden waren.

Damit es aber überhaupt zur langfristigen Etablierung einer solchen Arbeitsgruppe kommen konnte, reichten wir im Herbst 2012 eine Beschlussvorlage zur Gründung einer übergreifenden Arbeitsgruppe „Mittelbau“ im Fach Wissenschafts‑, Medizin- und Technikgeschichte bei unseren Fachgesellschaften ein. Als wir unsere Forderungen auf den jeweiligen Mitgliederversammlungen präsentierten, stießen wir zwar grundsätzlich auf viel Zustimmung, zum Teil schlug uns aber auch unverhohlenes Unverständnis entgegen. Dies wohl auch, weil die Annahme bestand, es ginge nur um die Aufstiegschancen des „Nachwuchses“ und unsere Initiative diene dem eigennützigen Zweck, „innerhalb der Hierarchie eines der wichtigsten Verbände Europas“ aufzusteigen, wie Heiko Stoff von seinen Erfahrungen mit gewissem ironischem Unterton berichtete.^1^ Manche – insbesondere ältere Kollegen – waren der Meinung, der Initiative der Aufmüpfigen werde schnell die Luft ausgehen.

Im Ergebnis konnte sich noch im Jahr 2012 die AG Mittelbau als übergreifende Arbeitsgruppe „Mittelbau der Medizin‑, Technik- und Wissenschaftsgeschichte“ der Deutschen Gesellschaft für die Geschichte der Medizin, Naturwissenschaft und Technik (DGGMNT), der Gesellschaft für Wissenschaftsgeschichte (GWG) und der Fachverbände für Wissenschaftsgeschichte und für Medizingeschichte konstituieren – ab Frühjahr 2013 auch der Gesellschaft für Technikgeschichte (GTG). Ein erstes gemeinsames Treffen fand am 16. November 2012 statt.

Der kleine Braunschweiger Kreis erweiterte sich schnell um die auf den Mitgliederversammlungen gewählten Vertreter:innen und weitere Interessierte. Für die DGGMNT waren dies anfangs Christine Wolters, Timo Engels und Florian Schmaltz; für die GWG Heiko Stoff, Bettina Bock von Wülfingen und Florence Vienne; Heiko Weber für den Fachverband Wissenschaftsgeschichte; Nina Lorkowski und Heike Weber für die GTG; Axel Hüntelmann für den Fachverband Medizingeschichte sowie außerdem Marion Hulverscheidt, Marion Steiner, Ulrike Thoms, Martina Schlünder und Alexander von Schwerin.^2^ Seit November 2014 war die AG Mittelbau auch bei Sitzungen des Nationalkomitees International Union for the History and Philosophy of Science als ständiges Mitglied präsent.

Als erstes veranstaltete die so frisch aufgestellte AG ein öffentliches Gespräch mit Andreas Keller, Leiter des Vorstandsbereichs Hochschule und Forschung der GEW, am 4. September 2013 an der HU Berlin. Im Lauf des Jahres 2014 schlossen sich die Mitgliederversammlungen von DGGMNT, GWG, GTG und des Fachverbands Medizingeschichte den „Empfehlungen für eine Stärkung des Mittelbaus: Gute Arbeit in der Wissenschaft“ der AG an. Das Verständnis für die Problematik und das Anliegen der AG hatte spürbar zugenommen, nicht zuletzt durch die entschiedene Unterstützung aus den Reihen der Mitgliedschaft und der Vorstände der Gesellschaften. Es wurde nun nicht mehr nur von der Förderung des Nachwuchses, sondern auch von der Notwendigkeit gesprochen, die Situation des Mittelbaus durch hochschulpolitisches Engagement zu verbessern. In den Empfehlungen der DGGMNT hieß es zum Beispiel:

„Weit mehr als ‚Nachwuchs‘ ist der Mittelbau eine zentrale Säule von Forschung und Lehre an den deutschen Hochschulen und Forschungseinrichtungen. […] Die DGGMNT betrachtet die Belange und Förderung des Mittelbaus als eine ihrer wichtigen Aufgaben. Die DGGMNT unterstützt Initiativen, Verantwortlichen in Hochschule und Forschungseinrichtungen Möglichkeiten aufzuzeigen, in ihrem Rahmen tätig zu werden.“^3^

In der von der DGGMNT neu begründeten Reihe „Junge Perspektiven“ veranstaltete Heike Weber 2014 einen Workshop zu Geschlechterverhältnissen in Wissenschaft und Technik. Über zwanzig Treffen der AG folgten bis zum Beginn der Covid-19-Pandemie – vor allem in Berlin, an Instituten, mal beim Gen-ethischen Netzwerk oder privat, besonders in der gemütlichen Wohnung von Bettina Bock von Wülfingen. 2018 – also schon bevor pandemiebedingt die meisten Treffen virtuell stattfanden – begann die AG damit, ihre Treffen online durchzuführen, da sich die teils teure Anreise nach Berlin für prekär beschäftigte Kolleg:innen als große Hürde erwies. Diese Entscheidung trug dazu bei, dass der Kreis der Aktiven seither tendenziell stabiler und größer wurde. Ein Newsletter zu aktuellen wissenschaftspolitischen Entwicklungen und Diskussionen half beim Austausch untereinander. Der im April 2023 verstorbene Heiko Weber orchestrierte aus Göttingen per Mail Vor- und Nachbearbeitung der Treffen mit guten Einfällen und Durchhalteparolen, nicht zuletzt aus seinen profunden Liedgutkenntnissen. Zu denjenigen, die sich aus der Ferne und dem Umfeld der AG einschalteten, gehörte der mitunter nicht zu Unrecht mit dem Fortgang der Dinge ungeduldige Christian Forstner – auch er ist im Juli 2022 viel zu früh verstorben.

An den Verhandlungen zur Fusion von DGGMNT und GWG beteiligte sich die AG Mittelbau durch Axel Hüntelmann mit dem Ergebnis, dass zu den Aufgaben der neuen Gesellschaft künftig gehörte, sich für „Chancengleichheit und die Belange des wissenschaftlichen Nachwuchses und des Mittelbaus“ einzusetzen.^4^ Ein Viertel der Mitglieder des Vorstands sollten künftig dem Mittelbau angehören. Auf der Gründungsversammlung der Gesellschaft für Geschichte der Wissenschaften, der Medizin und der Technik (GWMT) im Jahr 2016 wählte die Mitgliederversammlung erstmals Vertreter:innen für den Nachwuchs und den Mittelbau als Beisitzer:innen in den Vorstand, die zugleich die AG koordinierten (Nadine Metzger und Alexander von Schwerin). Diese enge Verbindung zwischen der AG Mittelbau und dem Vorstand der GWMT setzte sich fort, als David Freis, der die AG zusammen mit Christian Sammer seit 2018 mitkoordinierte, 2020 als Beisitzer für den Mittelbau in den Vorstand der GWMT gewählt wurde. 2023 übernahm Christian Zumbrägel dieses Amt.

Die AG verlegte sich in der Folgezeit auf die Sondierung der Lage, die Organisation von Diskussions- und Inforunden auf den Jahrestagungen sowie die Durchführung von Institutsumfragen, die das Ausmaß der Befristungen in den Fächern der GWMT dokumentieren und in ihrem Verlauf sichtbar machen sollten. Im September 2015 veranstaltete die AG erstmals eine Podiumsdiskussion zur Lage und Zukunft des Mittelbaus im Rahmen der gemeinsamen Tagung von DGGMNT und GWG an der TU Berlin, an der Vanessa Adam vom Deutschen Hochschulverband, Anke Burkhardt vom Institut für Hochschulforschung an der Universität Halle, Rainer Hansel von der GEW Berlin und Mechthild Koreuber von der Bundeskonferenz der Frauen- und Gleichstellungsbeauftragten an den Hochschulen teilnahmen. In den Jahren 2016 und 2017 folgten ähnliche Veranstaltungen zum Thema „Das novellierte Wissenschaftszeitvertragsgesetz im Praxistest befristeter Anstellungsverhältnisse“ mit Peter Ullrich vom *Netzwerk für Gute Arbeit in der Wissenschaft* (NGAWiss) beziehungsweise „Der Mittelbau organisiert sich (nicht). Was können wir bewegen?“ unter anderem mit Mathis Nolte vom Interdisciplinary Network for Studies Investigating Science and Technology (INSIST). Seitdem haben der Runde Tisch der AG und damit auch die unterschiedlichen Facetten der politischen Belange des Mittelbaus von Mental Health bis alternative Karrierewege einen festen Platz auf den Jahrestagungen der GWMT gefunden. Zusätzlich findet auch bei dem im Umfeld der Jahrestagungen stattfindenden Treffen des Driburger Kreises eine Vorstellung der AG statt.

Seit dem Jahr 2017 entstanden immer neue Initiativen an den Hochschulen mit ähnlichen Beweggründen und Zielen, wie wir sie in der AG Mittelbau verfolgten. Mit dem NGAWiss etablierte sich ein bundesweiter Zusammenschluss von Mittelbauinitiativen in deutschen Hochschulen und Fachgesellschaften. Die Mittelbau AG trat dem NGAWiss Anfang 2018 bei. Florence Vienne und Susanne Doetz engagierten sich im Koordinationskreis des Netzwerkes und insbesondere für die Belange prekärer ausländischer Wissenschaftler:innen und Exilwissenschaftler:innen in Deutschland.^5^ Zu Beginn des Jahres 2019 nahm das NGAWiss die Verhandlungen zur Neufassung des Hochschulpaktes zum Anlass, ein Aktionsbündnis mit den Gewerkschaften Verdi und GEW zu schmieden, aus dem die Kampagne „Frist ist Frust“ entstand. Mehr als 15.000 Unterschriften wurden für die gleichnamige Petition gesammelt. Die Hauptforderung war, die Mittel des neuen Hochschulpaktes in Zukunft vollständig und verbindlich für zusätzliche Dauerstellen mit fairem Lehrdeputat einzusetzen. Dank des Engagements der AG Mittelbau gehörten unsere Fachgesellschaften und Fachverbände zu den ersten Unterstützern der Kampagne; auch bei den Protestaktionen vor dem BMBF am 5. April und 2. Mai 2019 waren Mitglieder der AG dabei. Allerdings konnte die Kampagne nicht erreichen, dass das BMBF verbindliche Vorgaben für die Schaffung von Dauerstellen in den drei Pakten verankerte, die zur Sonderfinanzierung von Lehre und Forschung beschlossen wurden.^6^ Mehr Wucht entfaltete kurze Zeit später der dezentrale Protest #IchBinHanna (und später mit intersektionaler Perspektive #IchbinReyhan oder mit Unterstützung „von oben“ #ProfsFuerHanna), der von Politik und Medien stärker wahrgenommen wurde und an den das folgende aktivistische Engagement von NGAWiss, GEW und Verdi anknüpfen konnte. Gleichzeitig hat das NGAWiss seit 2020 mit der geballten Fachkenntnis seiner Mitglieder zum einen Vorschläge für alternative Beschäftigungsmodellen erarbeitet, die kostenneutral zum Wohle aller Mitarbeitenden an deutschen Hochschulen umgesetzt werden könnten (2020),^7^ zum anderen den Effekt der letzten Novelle des Wissenschaftszeitvertragsgesetzes auf die realen Beschäftigungsbedingungen wissenschaftlich evaluiert (2022).^8^ Auch über diese Evaluation sprach der daran maßgeblich beteiligte Soziologe und NGAWiss-Aktivist Tilman Reitz, als er Gast des von Laurens Schlicht organisierten Runden Tisches der AG 2022 in Erfurt war.

Neben der politischen Dimension des Mittelbau-Aktivismus fokussierte die AG in den letzten Jahren insbesondere auf wenig diskutierte Themen wie psychische Gesundheit und alternative Karrierewege in der und aus der Wissenschaft, aber auch den Arbeitsalltag durchziehende Fragen zu Machtmissbrauch und diesbezüglichen Unterstützungsmöglichkeiten. Aktuell wurde die AG vom Vorstand der GWMT damit beauftragt, die Aufgaben einer neu einzuführenden Vertrauensperson zu entwerfen, die Mitgliedern des Mittelbaus bei Konflikten eine stärkere Stimme zu geben vermag.

Die Kernforderungen unseres Positionspapiers „Für die Überwindung des deutschen Sonderwegs der Nachwuchsuniversität“ von 2014 sind zehn Jahre später leider immer noch aktuell. Dies zeigt nicht zuletzt die Beschäftigungsstatistik in unseren Fächern, welche die AG Mittelbau alljährlich seit 2017 präsentiert, nachdem es nach einigen Mühen gelungen war, erstmals Daten „zur Lage des Mittelbaus in unseren Fächern“ zu erheben. Für 2017 konnten die Daten von 43 Instituten ausgewertet werden. Eines der Ergebnisse lautete, dass 13 Prozent der Mittelbaustellen an den 43 Instituten unserer Fächergruppe unbefristet und 87 Prozent befristet sind. Dies entsprach in etwa dem bundesweiten Durchschnitt jenseits der Wissenschafts‑, Medizin- und Technikgeschichte. Die letzte Umfrage für das Jahr 2022 hat eine kaum veränderte Befristungsquote von 84 Prozent ergeben und erinnert schmerzlich daran, dass sich die prekäre Beschäftigungssituation des überwiegenden Teils der Kolleg:innen im Mittelbau in den letzten Jahren trotz des gesteigerten Bewusstseins für die Problematik keinesfalls geändert hat. Die Abschaffung der „deutschen Nachwuchs-Universität“, die wir seit 2013 laut einfordern, sehen wir nicht nur als ein sozialpolitisches Gebot. Sie ist auch eine unabdingbare Voraussetzung für die Verbesserung der Qualität von Lehre und Forschung in Deutschland. Jetzt liegt es „nur noch“ am fehlenden politischen Willen, das Wissenschaftssystem nachhaltig zugunsten aller Beschäftigten zu verändern. Die kritischen Stellungnahmen zu den Referent:innenentwürfen der Novelle des Wissenschaftsvertragszeitgesetzes, die unsere Fachgesellschaften und Berufsvereine auf Initiative der AG Mittelbau hin abgegeben haben, bleiben (noch) ungehört.

Die AG Mittelbau waren oder sind: Bettina Bock von Wülfingen, Fritz Dross, Julia Engelschalt, David Freis, Eike Christian Heine, Axel C. Hüntelmann, Elena Kunadt, Matthis Krischel, Nina Lorkowski, Nadine Metzger, Christian Sammer, Florian Schmaltz, Alexander von Schwerin, Katharina Seibert, Felix Sommer, Heiko Stoff, Florence Vienne, Christine Wolters und viele hier ungenannte Aktive.

## Footnotes

1 So in der Diskussion auf der Mitgliederversammlung der GWG; Heiko Stoff an Florence Vienne, Bettina Wahrig und Alexander von Schwerin, 30.09.2012.

2 Laut Anwesenheitsliste, Protokoll Treffen Mittelbau, 16.11.2012 und E‑Mail-Korrespondenz.

3 Empfehlungen der DGGMNT für eine Stärkung des Mittelbaus: „Gute Arbeit in der Wissenschaft!“ vom 12. September 2014, Nachrichtenblatt der GWMT, Nr. 1 (2014), S. 49–51, hier: 50. Siehe auch Protokoll der Mitgliederversammlung der der Deutschen Gesellschaft für Geschichte der Medizin, Naturwissenschaft und Technik e. V. am 12. September 2014, München, Nachrichtenblatt der DGGMNT, Nr. 2 (2014), S. 8–26, hier: 23.

4 Hier und nachfolgend § 2 bzw. § 9 der Satzung der Gesellschaft für Geschichte der Wissenschaften, der Medizin und der Technik (GWMT) e. V. in der von der Mitgliederversammlung am 22.04.2016 beschlossenen Fassung, Nachrichtenblatt der DGGMNT, Nr. 1 (2017), S. 6–13, hier: 6 bzw. 10.

5 https://mittelbau.net.

6 Dazu auch Elena Kunadt und Florence Vienne: 7 Jahre Hochschulpolitisches Engagement der AG Mittelbau in der Wissenschafts‑, Medizin- und Technikgeschichte: Vom Kampf gegen die „Nachwuchs-Universität“ zur Kampagne „Frist ist Frust“, GWMT-Newsletter, Nr. 01 (2019), S. 8–10.

7 https://mittelbau.net/diskussionspapier-personalmodelle-2/diskussionspapier-personalmodelle/.

8 https://mittelbau.net/gemeinsames_statement_wisszeitvg_ii/.

